# Family Separation and the Impact of Digital Technology on the Mental Health of Refugee Families in the United States: Qualitative Study

**DOI:** 10.2196/14171

**Published:** 2019-09-03

**Authors:** Sayyed Fawad Ali Shah, Julia Meredith Hess, Jessica R Goodkind

**Affiliations:** 1 Department of Communication Jacksonville State University Jacksonville, AL United States; 2 Department of Pediatrics University of New Mexico School of Medicine Albuquerque, NM United States; 3 Department of Sociology University of New Mexico Albuquerque, NM United States

**Keywords:** refugees, family separation, mental health, mobile phone

## Abstract

**Background:**

Conflicts around the world have resulted in a record high number of refugees. Family separation is a critical factor that impacts refugee mental health. Thus, it is important to explore refugees’ ability to maintain contact with family members across the globe and the ways in which they attempt to do so. It is increasingly common for refugees to use information and communication technologies (ICTs), which include mobile phones, the internet, and social media sites, such as Facebook, WhatsApp, Skype, and Viber, for these purposes.

**Objective:**

The aim of this study was to explore refugees’ perceptions of the impact of communication through ICTs on their mental health, the exercise of agency by refugees within the context of ICT use, especially their communication with their families, and logistical issues that affect their access to ICTs in the United States.

**Methods:**

We used a constructivist grounded theory approach to analyze in-depth interviews of 290 adult refugee participants from different countries, who were enrolled in a randomized controlled trial of a community-based mental health intervention.

**Results:**

Analyses showed that communication through ICTs had differing impacts on the mental health of refugee participants. ICTs, as channels of communication between separated families, were a major source of emotional and mental well-being for a large number of refugee participants. However, for some participants, the communication process with separated family members through digital technology was mentally and emotionally difficult. The participants also discussed ways in which they hide adversities from their families through selective use of different ICTs. Several participants noted logistical and financial barriers to communicating with their families through ICTs.

**Conclusions:**

These findings are important in elucidating aspects of refugee agency and environmental constraints that need to be further explicated in theories related to ICT use as well as in providing insight for researchers and practitioners involved in efforts related to migration and mental health.

## Introduction

### Background

Conflicts in different parts of the world, especially the Middle East and Africa, have resulted in a significant number of people who have experienced forced displacement worldwide. According to the United Nations High Commission for Refugees (UNHCR) [1], the number of displaced people reached an all-time high—70.8 million—at the end of 2018. UNHCR has classified 25.9 million of these displaced people as refugees. UNHCR defines a refugee as someone who has been forced to leave his or her country because of violence, persecution, or war and has a well-founded fear of persecution that prevents them from returning home [2].

Refugees, similar to other forcibly displaced people, often experience traumatic events, such as torture, mass killings, and political victimization. Exposure to these traumatic experiences negatively affects the mental health of refugees [3,4]. In addition to witnessing and experiencing these traumatic events, refugees often leave some or all of their family members behind. Family separation is a critical factor that impacts the mental health of refugees [5]. Often, refugees use information and communication technologies (ICTs), which include mobile phones, the internet, and social media sites, such as Facebook, WhatsApp, Skype, and Viber, to stay in touch with their family [6-8]. This study aimed to understand (1) refugee perceptions of the impact of the use of ICTs to communicate with family members on their mental health and (2) the exercise of agency by refugees, within the context of their imagined affordances of ICTs (users’ perceptions of communication technologies that influence how they approach them), especially with respect to their communication with their families.

### Family Separation and Mental Health

Several studies have documented the negative impacts of family separation on the mental health of refugees [5,9]. Sudanese refugees resettled in Australia perceived family separation as the most significant factor that impacted their mental well-being. They described their most salient mental health issues as resulting from missing their families and being concerned about the financial situation of their families who were residing in Sudan [10]. Miller et al [9] conducted a mixed-methods study to understand how family separation affected the mental health of refugees who resettled in the United States. The authors found that separation from a family member or loved one was 1 of only 2 traumatic experiences out of the 27 they measured, which explained significant variance in all the 3 components of refugees’ mental health included in their study—posttraumatic stress disorder (PTSD) symptoms, emotional distress, and psychological quality of life—even after accounting for refugees’ overall level of trauma exposure. They also found that family separation caused mental stress among refugees in different forms. For example, refugees were fearful about the safety of their family members who remained in their home countries, which made them question their decision to resettle in the United States.

### Information and Communication Technology Use Among Refugees

ICTs are often very important in the lives of refugees. ICTs can facilitate refugees’ well-being by helping them navigate the difficult terrain to their destinations, accumulate information about host countries, and get in touch with friends and family [11-14]. However, ICT use may also endanger refugee lives by making their information available for law and order personnel in the countries of their destinations or by making state or nonstate actors of the countries they are fleeing from aware of their flight or location [15,16]. Studies looking at refugees’ use of ICTs are mainly focused on (1) refugees’ mobilization from their home countries to their countries of refuge [14,15,17] and (2) the resettlement of refugees in new countries [6,11,13,18-20].

Studies investigating the use of ICTs primarily explore their use during the journey: maps to navigate routes, text and phone to remain in contact with family and their smugglers, and at times to save their lives by calling the border patrol in case of emergency [14,15]. Similarly, several studies examine the ways in which refugees use ICTs to meet several social and economic needs once they resettle in their destinations. For example, Kaufmann [21] found that Syrian refugees in Vienna used smartphones to access information, learn a language, and stay in touch with their families. He suggests that the refugees in his study were emotionally attached to their smartphones. Similarly, Wilding [22] noted that young refugees in Australia used ICTs to maintain their links with several communities at a time, including family and friends in the country of origin; therefore, the ICTs helped them transcend time and space. A study done in the Netherlands found that refugees used ICTs to learn local language and culture, among other things [11]. Similarly, studies have noted that refugees use ICTs to access different resources. For example, a study in Kenya revealed that ICTs offered platforms for refugees to access higher education in refugee camps [23]. The studies that examined the use of digital technologies by refugees during resettlement mainly focus on the use of ICTs for gratifying social needs, such as communicating with other refugees, family and friends, and promotion of health [20,24,25].

### Information and Communication Technologies and Family Separation

Given the distress that family separation causes to refugees, it is important to explore refugees’ ability to maintain contact with their family members and the ways in which they attempt to do so. An increasingly common way through which refugees are able to stay in touch with family members is through the use of ICTs. ICTs enable *copresence*, a state of being emotionally connected with families that are far away [26].

ICTs can act as a vital lifeline for refugees who have resettled in a new country, and research has revealed that ICTs can help improve the mental health of refugees by enabling them to find displaced family members, communicate with their families and friends and inform them of their needs, and receive and send financial assistance [25]. UNHCR and host country governments are also exploring the use of ICTs to help refugees resettle in host countries and reduce the distress caused by family separation. Some research suggests that ICTs can contribute to bridging the separation gap by making Web-based family reunification possible [10]. Thus, it seems that digital technology can provide separated families the opportunity to retain a communication link with their families.

Recent studies have found that cellular networks are the most common tools used to access the internet among refugees and that refugees living in camps use the internet and social media for several purposes, including connecting with family, building social support networks, and telling their stories [27,28]. A study conducted in a refugee camp in Syria noted that social media, such as WhatsApp and Facebook, were more often used for communication and information-seeking purposes by refugees than mobile voice, short message service text messaging, Skype, and email [29]. Digital technology has not only helped refugees connect with their families, but it has also helped them integrate into their host countries by allowing them the opportunity to access amenities on the Web, such as banking systems and aid agencies, and share their stories with different segments of the society [30]. Overall, research has documented that the primary use of ICTs among refugees is for communicating with their families [31]. However, the literature on the impact of ICTs on the mental health of refugees in the context of family separation is limited.

### Refugees’ Agency in the Usage of Information and Communication Technologies

Research shows that refugees’ agency to interact with or through ICTs is dependent on the imagined affordances of the ICTs [16]. Imagined affordances are the users’ approaches to a technology shaped by the users’ expectations from that technology [32]. For example, in the context of mobility to Europe from Syria, refugees used smartphones to navigate their routes and stay in touch with their families and, at the same time, they feared negative consequences, such as being exposed to surveillance by state and nonstate actors [16]. Therefore, these possibilities of benefit and harm shaped how refugees strategically used technology while moving to their destination. However, there is a lack of literature on how refugees make strategic decisions about and exercise their agency to communicate through ICTs with their friends and family, especially in the context of resettlement in host countries.

### Barriers to Refugee Usage of Information and Communication Technologies

Some scholars argue that even though ICTs are essential tools of communication among refugees, the limited ability to physically access ICTs and limited skills to use these technologies have resulted in inequities among different refugee groups [7,33]. For example, newly arriving refugees to Australia have shown low ICT literacy, as they were deprived of Web-based communication tools during their displacement [28]. As the number of forcibly displaced people around the world increases, it is essential to understand more about barriers to ICT use, which is faced by diverse refugee populations.

Thus, although there is burgeoning and important literature on ICT use among refugees, there is a need to explore refugee perceptions of the impact of ICTs on their mental health and how refugees’ decisions and actions illustrate their agency in their interaction with ICTs, in the context of resettlement in their host countries, and potential barriers they may face to ICT usage.

Hence, our study was guided by 2 overarching research questions.

Research question 1: What is the range of mental health effects refugees report as a result of communicating with separated family members using ICTs?

Research question 2: Given the potential positive and negative impacts of ICT use on refugee mental health, how do refugees exercise agency (within a context of opportunities and constraints that shape their access to ICTs) to protect their well-being?

## Methods

### Setting

The data for this study were collected as part of a randomized controlled trial of the Refugee Well-being Project (RWP), from 2013 to 2018. Funded by the National Institute on Minority Health and Health Disparities, the study examined the effectiveness of a community-based advocacy and learning mental health intervention for recently arrived refugees in New Mexico, 1 of 49 states where refugees are resettled in the United States. Participants included refugee adults from Afghanistan, Iraq, Syria, and the Great Lakes region of Africa (Burundi, Democratic Republic of Congo, and Rwanda), who were resettled in the United States for less than 3 years. Participants were randomized by household to an intervention or waitlist control condition. Participants in the intervention were matched with an undergraduate or graduate student (receiving training and supervision through enrollment in a 2-semester course) at the University of New Mexico. Together, the student and refugee partners engaged for 6 months in mutual learning and the mobilization of community resources to address the goals and unmet needs of refugee participants. Those in the waitlist control group were invited to participate in a 1-time stress management session, and they were eligible to participate in the RWP intervention after the study period ended. All participants who reported high PTSD symptoms were invited to receive Narrative Exposure Therapy (NET) at no cost.

### Participants

Study participants were enrolled in 4 cohorts (2013-2016). Of the 290 study participants, 31.0% (90/290) were from the Great Lakes Region of Africa, 36.2% (105/290) were Afghan, and 32.8% (95/290) were Middle Eastern (Iraqi and Syrian). A total of 52.4% (152/290) of the participants were women, and they ranged in age from 18 to 71 years (mean 34.6). More than half (58.3%; 169/290) of the participants were married, with 33.1% (96/290) single, 6.6% (19/290) widowed, and 1.7% (5/290) divorced. At the first interview time point, they had been in the United States for an average of 30 weeks. Participants completed mixed-method interviews at 4 time points (pre, mid, post, and follow-up), over the course of 12 months. All participants completed a qualitative interview at the first time point; we purposely sampled 16 participants in each of the 4 cohorts for qualitative interviews at the 3 later time points, selecting participants who presented rich preliminary data in both the NET-eligible (clinically significant PTSD symptoms) and ineligible categories and across gender and national origin groups.

### Methodological Approaches

The mixed-method research used a convergent parallel design [34]. The qualitative and quantitative strands were separate; however, we designed each strand with complementarity at the forefront, meaning that we built on the strengths of each strand to answer specific kinds of research questions. The data reported in this study are qualitative, which were collected with an aim to provide an in-depth understanding of processes we were seeking to understand. Constructivist grounded theory guided the qualitative analysis [35]. Constructivist grounded theory acknowledges that analysis is a coconstruction of researchers and participants and, as such, is an interpretivist approach, but one that takes into account the specific place and time in which the research is conducted. Constructivist grounded theory is flexible, but in general, its tenets direct researchers to stay close to the data, attend to processes and actions, and use abduction to make inferences about the social world with which we are engaging.

The qualitative component of each interview involved a semistructured interview guide, with questions in numerous domains, including open-ended questions about the benefits and challenges of life in the United States, work experiences, and impacts of resettlement on participants’ health and family. Other domains in the interview included questions about refugees’ social networks, cultural maintenance, and changes across all domains since the previous interview. The quantitative strand of the study included collecting information about participants’ mental health, social networks, and access to resources. Data from previous RWP research had underscored the importance of refugee participants maintaining communication and receiving and providing support from family members and friends from whom they had been separated during the migration journey. Thus, we added questions about if and how often they contacted family members and others, as well as the impacts these contacts had on their health and well-being.

Interviews were conducted with an interpreter who spoke a common language with the participant (eg, Arabic, Dari, Pashto, Kirundi or Kinyarwanda, Swahili, or French). The average interview length was 50 min, with a range from 12 to 143 min. Participants were provided with gift cards in compensation for their time (amounts increased for each time point from US $20 to US $50). The research was approved by the University of New Mexico Human Research Protections Office.

### Data Analysis

English portions of qualitative interviews were checked for accuracy, anonymized, and imported into NVivo 10, a qualitative data analysis software package (QSR International). All qualitative data were analyzed using a multistep process. First, all data were coded according to question, allowing for rapid analysis of research questions. To begin our analysis for this study, we focused on participant responses to the following questions: “Are you still in contact with people from your home country or country of first refuge?” and “How does that impact your well-being?” A second round of coding was applied to the dataset by 2 independent coders. The hierarchical coding tree was collaboratively established around inductive and deductive codes that were primarily descriptive in nature. Given the large number of interviews and resulting number of references coded at prominent categories (eg, health), we used NVivo’s querying functions to examine intersecting codes. Thus, we looked across the entire dataset for themes related to mental health that intersected with participant discussions of family in their home country or country of refuge (see Table 1). This was achieved through using the matrix query function of NVivo 10.

**Table 1:** Number of themes related to mental health, which intersected with participants’ discussions of their social networks.

Health nodes and subnodes		Number of health nodes cocoded at the node *Social network—family, friends, neighbors, and colleagues*
**Health**		14
	Healing, recovery	03
	Memory, forgetfulness	01
**Mental health**		21
	Afraid	07
	Alone, lonely	09
	Depressed, depression	11
	Happy	42
	Homesick, missing family and friends	75
	Positive, optimistic outlook	18
	Sad	20
Stress		27
Suffering		07
Tired		04
Worry		52
Physical health		14
Treatment		04

Third, focused coding and memo writing [35] were conducted on prominent categories related to communication through ICTs with family and its impact on well-being. Memo writing attempts to (1) determine the definition of a category, explicating its subcategories; (2) examine the conditions under which the category arises, is maintained, and changes; (3) describe its consequences; and (4) show how it relates to other categories. The data were also examined for anomalies and contradictions to explore further in the analysis. Sorting, diagramming, and elucidating connections and relationships among these ideas were also done through memo writing [35]. These memos were the basis of the results shared below.

## Results

Refugee participants described a range of mental health effects from communicating with separated family members using ICTs, including positive impacts, such as healing, encouragement, social support, and bridging the physical distance separating them, and negative effects related to distress, elicited by heightened concerns about family’s safety and feelings of helplessness, as they could not help their distant family members.

### Communication as a Healing Process

Data from the semistructured qualitative interviews revealed that ICTs, as channels of communication between separated families, were a major source of emotional and mental well-being for a large number of refugee participants. Many participants described the process of talking to their families through ICTs as mentally, emotionally, and spiritually healing. They believed that talking to their families and friends through phones, social media, and other services, such as WhatsApp and Viber, made them feel good and relieved stress and depression. For example, an Iraqi man explained the following:

There is no day can pass without talking to them. I have to talk to them every day and all the times, a lot of times.

Thereafter, the interviewer asked the following question:

And when you talk with them how does that make you feel?

The participant replied by saying the following:

Of course, I will be comfortable, I will be comforted, because whenever I feel depressed, and a boring time, I have to talk to them, I have to call them.

### Encouragement and Social Support From Family and Friends

Participants discussed the encouragement and moral help they received from talking to friends and family members on several occasions. They described the encouragement they obtained from their family and friends to address the challenges they face in the United States as helpful in managing postresettlement stressors. In part, this was because communication with family and friends prompted the participants to think about the financial and political situations of their home countries. These reminders from families and friends motivated them to face the difficulties of resettlement and living in the United States. Communication with family and friends also provided them with the much-needed social support required to persevere in challenging times. A male participant from Iraq described how talking with people from home affected him:

Yeah, I’m still like in contact with my friends in Syria but just as far as saying hi and stuff. My friends are like encouraging me and telling me to be patient, friends from over there tell me to be patient, that nothing comes as simple as you want it to be.

Then, the interviewer asked the following question:

And so, when you talk with your friends and family in Iraq and Syria, how does it make you feel and how does it affect your well-being?

The participant said the following:

In a good way. They make me feel more comfortable spiritually, like deep inside of me. I talk to them over Tango, and I sit with a bunch of them talking, and they just make me feel a lot better and happier.

The comparison of the financial, political, and educational opportunities available for participants and their dependents in the United States and their families and friends in the home countries also motivated participants to stay in the United States, despite facing many problems associated with resettlement. A male participant from Democratic Republic of Congo said the following:

When I talk to them, I always compare my life and their lives, so I remember where I came from. I know they have so many problems. They don’t have food. They don’t have money to pay rent. They don’t have money to take their kids to school. They have all those kinds of problems. Then, I thank God for bringing me here. I don’t need food, and I don’t need to pay for school for my kids. I thank God for that even though I know that I do have problems. I haven’t found a job and other stuff, but there’s a difference between them and me.

Communication with family and friends reminded participants how hard things were back home, which encouraged them to get through tough times in the United States.

### Overcoming the Physical Distance

Another prominent theme in our data was how ICTs helped bridge the gap between separated families. Participants described how their communication with relatives through ICTs helped decrease the burden of separation from their families and fulfilled some of their emotional and mental needs by making them feel at home when they were away from their homes. An Afghan woman described this process:

I say every night I talk to them. At least like I think they’re very close to me. I say we talk on the Skype; they see us like we feel like they are very close and in touch with us.

An Iraqi woman also echoed these feelings:

When I talk with them I feel good. You know, the day passes very happy for me and I feel that I’m home and I feel that everybody’s around me. Nowadays the networking system [ICTs] has really brought the people very close to each other.

Another Iraqi participant echoed the same feelings:

Yeah, I have my friends and I usually contact them like to see what things over there are happening. What things changed after we left? Yeah, so we’re in continuous contact with them.

Then, the interviewer asked the following question:

And so, how does that make you feel being able to contact them still?

The participant responded by saying the following:

It makes me feel better because when you leave something that you live for a long time, you think that there is something that attracts you. So, you need to know what happened over there. Who is feeling well. Who is feeling not well. Yeah so, when you are in continuous contact with your friends, with your family, it will support you here.

The comment shows the ways in which communication among families, through ICTs, motivated participants to overcome the difficult circumstances in their host countries, with the help and support of faraway friends and family. However, not all communication with family members was helpful or had a beneficial effect on participants. Some refugees described the process of communicating with their separated family members as depressing and stressful.

### Communication as a Source of Depression and Stress

#### Distress of Leaving Family Members in Conflict Situations

In some interviews, participants stated that they felt stressed and depressed after talking to their family members and hearing about the dangers they faced every day. A female participant from Afghanistan felt that she had done injustice to her family members by leaving them back home.

The interviewer asked the following question:

When you talk to them, how does that impact your well-being?

The participant responded by saying the following:

My [daughter-in-law’s mother] and family members are crying when I call, and I cry also. I’m the most unlucky woman in the world, so I don’t show this to my son because he is here working hard, and I don’t want him to know how badly I feel. So, I hide it. When we came from Kyrgyzstan to the U.S., we stopped in Istanbul and then went to Chicago, and I cried the whole way because I had to leave my son there.

Another female participant from Democratic Republic of Congo spoke about how she felt emotionally drained after talking to her family members through the phone or internet.

The interviewer asked the following question:

Yeah? Okay. And is that difficult for you? Is that, sometimes it can be positive and sometimes it can be negative because you don’t know how people are doing, and I’m just wondering how that’s been for you?

The participant responded by saying the following:

We’re not okay because it’s hard especially when you leave your family somewhere and you know that place is not good. They call us and tell us to check the social media and we check Facebook and see shootings and people are dying. So, it’s not easy.

These participants hoped that their stress would be relieved after taking refuge in the United States, but talking to their families and seeing and hearing about their ordeals resulted in increased emotional distress. Hearing troubled and fearful voices of their relatives and seeing videos of their houses and streets through apps, such as WhatsApp, Skype, and Viber, reminded many participants of the difficulties of their past and the difficulties their loved ones still faced.

#### Helplessness

Several participants described how helpless they felt when they talked to their family members. They relayed that talking to their separated family members was disturbing, as they could talk to their families but could not help them out. For example, one of them, a woman from Syria commented.

The interviewer asked the following question:

How often are you in contact with your mom and your sister?

The participant replied by saying the following:

If I cannot contact her or call her within two days I’ll be crazy. She will be crazy, too.

Then, the interviewer asked the following question:

How does it impact your well-being to be in touch with your family?

The participant responded by saying the following:

Of course, it’s helping me a lot contacting her every day. Now she’s sick; she cannot leave the bed for two months now. I cannot go to help her. Of course, talking to her is kind of helping, but not going to her, being with her, serving her, taking care of her, as a sick mom, and when I went to her last time, she was sick also, but when she saw me she kind of refreshed herself, she was fine, she was happy. I wish I can go, but I’m really afraid. If I have a little money, I will go, but I’m really afraid that might hurt my status as a refugee or my green card. I don't know. But yeah, that, of course, will help, and of course talking to her every day is really kind of helping.

Several other participants discussed feelings of helplessness after talking to their relatives. For example, a woman from Iraq said the following:

When I speak to my family back home, I really get very worried about my mother especially because my mother is all the time telling me that she is sick because I am away from her and she is really worried about me. All the time, she would tell me that ‘You need to come back to Iraq no matter how difficult it is over here in Iraq. You will just live the way we live.’ I get concerned about what she’s telling me, that I lose my focus. Even when I take my English classes, my mind is all the time with my family members back home, and I all the time think about it.

The messages communicated in these exchanges were stressful for participants and increased the emotional distress of participants. The participants said that even though the ICTs helped them keep in touch with their families, the technology could not replace face-to-face communication, as their inability to physically care for their families was distressing.

### Refugee Agency and Information and Communication Technologies

Given the positive and negative impacts of ICT use on refugee mental health, it is important to understand how refugees exercise agency (within the context of opportunities and constraints that shape their access to ICTs) to protect their well-being.

We found that participants’ agency around ICT use was evident, as they satisfied their need to maintain relationships with their family members, including whether to remain in touch and how often. However, the use of ICTs also had unintended effects on refugees’ mental health. For example, participants described their own agency with respect to controlling communication from their side, but they could not control messages they received from their family members. Several participants said that the communication process with their separated family members through digital technology was mentally and emotionally draining.

### Communication to Hide Adversities

Interestingly, participants discussed the ways in which they used different ICTs to hide adversities from their family members. Some participants spoke about how they did not reveal their actual living conditions to their family members when they used digital technologies to communicate with their separated families. They described relatives thinking that they were living lavishly in the United States. However, family members from the country of origin were not familiar with the everyday struggles the participants faced in the new country. Many refugees covered up their struggles so as to not cause family members to worry. Strategic use of some ICTs over others made it possible for them to hide things they did not want to share. For example, a man from Iraq described how he used different communication methods:

Sometimes I talk to them by phone, so they just hear my voice. Sometimes I’m talking with them by the Facebook, as I said before, without any picture, but remember that whenever I’m talking to them like that, I pretend that I’m in the restroom or in the bathroom or something like that, to hide myself. My sister called me a few days ago and by the Viber. Have you heard about the Viber?

The interviewer asked the following question:

The app?

The participant responded by saying the following:

Exactly, because the picture is just like a video record, so I told my wife to talk to my sister and tell her I’m not in the home right now.

Then, the interviewer added the following:

I’m sorry. So how does that impact you and how you feel when you talk to your family?

The participant responded by saying the following:

Actually, I feel sad because they used to see me in a better situation, healthy. And if they see me like that right now I will feel very sad and uncomfortable, so that will affect my situation, my health.

The same feeling was shared by other participants, who explained that they did not share current difficulties with their families to protect their families and not cause worry, as their families were not able to help them at that time. Participants worried that telling their family members about the hardships they faced in the United States as refugees would further harm the physical and mental health of their faraway family members.

In summary, participants exercised agency to control the communication process with their separated families on the basis of the perceived outcome of the communication. Some participants did not share the problems they were facing in their country of refuge. For example, several participants described avoiding using phone cameras in their communication with their families to make their families believe that they were living a good life and to protect their families from seeing their stress. Agency in the use of ICTs reflects the differing ways participants tried to control the impact of communication with family and friends on their mental health.

### Financial and Logistical Barriers Restricting Access to Information and Communication Technologies

Financial and logistical issues limited some refugees’ agency with respect to their access to ICTs. Refugees had mixed experiences with ICTs in terms of the impact on their mental health and well-being; in addition, refugees’ access to ICTs also varied greatly. In particular, we found that certain financial and logistical issues restrained some refugees’ access to ICTs. A Burundian man described how his financial constraints limited his communication with his family:

Yeah, we are not in communication yet because we don’t have money to buy a phone card (the way that we call over there) because we don't have the money yet.

Some participants could not contact their families through the internet because of poor internet infrastructure in their home countries.

The interviewer asked the following question:

How often do you talk or do you Skype or Viber or whatever?

The participant from Afghanistan responded by saying the following:

Yeah, calling my family members and things it’s very hard because we have to make a phone call. I guess a $5 [prepaid calling card lasts for] 15 minutes, something like that you can talk through a phone. But you know, in Afghanistan not everybody got access to the internet. The phone technology...kind of new there. So some of them have...Facebook messenger, and they contact just with one. My brother, he got the internet on his phone. The only other people they have a net connection in their phone, those are interpreters, so I’m in contact with them.

A participant from Afghanistan said that when she and her family arrived in the United States, they did not know how to contact their family members through internet or phone. However, she subsequently learned how to get in touch with them:

Yeah, it’s very good for me when I talk to them and the first we didn’t know where to buy telephone cards, or we didn’t have the internet. It was so difficult, and I cried every night, that I couldn’t talk to the people there.

The time difference between the United States and the refugees’ home countries also served to limit communication among family members. A man from Iraq said that the time difference and the time constraints (because of his work) had caused strain between him and his family.

The interviewer asked the following question:

And how often do you talk to them?

The participant replied by saying the following:

When I have the time, and before I get to work, I was always in contact with them, and make conversation and ask for them. But with the time, with the work, I get a little bit and little bit and little bit. And they blame me [now for not calling them]. “Why don’t you contact us again like before?”

In summary, our qualitative results demonstrated that refugee participants experienced both positive and negative effects on their mental health from their communication with their families through ICTs; in addition, they attempted to moderate these effects through strategic decisions about their use of ICTs. Furthermore, participants described the lack of financial resources, poor information technology infrastructure in their home countries, and time constraints in the United States as the major hurdles for using ICTs to communicate with their families.

## Discussion

### Principal Findings

This study used in-depth qualitative interviews with 290 refugees from Afghanistan, Iraq, Syria, and the Great Lakes Region of Africa to understand how participants exercised agency in the usage of ICTs. Furthermore, we explored how their agency was shaped by imagined affordances—users’ expectations of ICTs and what actions are available to them as part of the interaction with those technologies that practically shape how users approach ICTs [32]. As such, this study provides important insight into refugees’ perceptions of how the use of digital technology or ICTs, such as mobile phones, social media, and other apps, such as WhatsApp and Viber, to keep in touch with family affects their mental health. We also explored the types of logistical problems that refugees face in accessing ICTs.

Our results show that refugees make strategic use of ICTs and that the exercise of their agency was based on the imagined affordances of ICTs or what refugees expected from ICTs. The primary motive for the use of ICTs among refugees was staying in touch with their separated families. ICTs helped them transcend physical boundaries. Our findings also elucidate that ICTs impacted individuals differently. Some participants described communication with their families through ICTs as “relieving,” “relaxing,” “tension-free moments,” “motivating,” and “encouraging”; others described communication with their families through ICTs as “depressing,” “stressful,” and “disheartening.” For some participants, hearing of their families’ struggles caused a feeling of helplessness, which was related to their inability to be there with them. Furthermore, some participants felt the need to keep from family members the stress of their everyday lives to not burden them with the same feeling of helplessness. These dynamics suggest that although ICTs have greatly improved the frequency of contact and the ability to keep in close touch, they may also exacerbate feelings of distress through increasing knowledge of the difficulties family members face, highlighting the feeling that one is not able to intervene to the desired extent. Another interesting aspect of ICTs is the ability participants have to see each other (rather than only hearing each other’s voices). We found that refugees decide whether to make a video call with their families on the basis of the imagined affordances of the video call. For example, we found that for some refugees, this facilitated feelings of closeness and connection, whereas for other refugees, the visual function of some ICTs added a layer of complication to communications when they did not want their family members to see them, as it would make it difficult to hide their poor health, distress, or unfavorable living conditions. In addition, some participants contended that they could not access ICTs because of financial issues and logistical problems.

### Theoretical Implications

Our study has implications for an important theory in the field of communication, Uses and Gratification Theory. The theory proposes that media users are active stakeholders in communication processes and that they actively seek media channels and content that gratify their needs [36]. The theory takes into consideration several aspects that are critical to our study, including a focus on the agency of users to choose how often and under what circumstances they communicate through a particular media outlet to gratify their needs. Although social theorists generally recognize agency—the ability to act in response to desire or motivation—as a human universal [37], refugees are often ascribed little or no agency, as the social constraints within which they live can be extreme [22]. Thus, it is important to recognize the spheres in which refugees employ agency in their everyday lives.

Gillespie and colleagues (2018) noted that the current application of Uses and Gratification Theory in the context of refugee migration is too simplistic, and they called for investigation of the fluid relationship between technology affordances and user agency in the context of refugee migration [16]. Our study addresses this call by exploring the relationship between user agency and the perceived outcomes of communication through ICTs. Our findings suggest that the exercise of agency by refugees in their interactions with ICTs was dependent on the imagined affordances of the ICTs [16,32]. Importantly, we found that refugees acted to use ICTs to gratify their needs of staying in touch with separated families and friends in their countries of origin, and we found that ICTs enabled them to transcend physical borders. The feelings of happiness among refugee participants who were able to remain connected with their separated families through ICTs emerged as a major theme. Participants discussed the importance of ICTs to their mental health, as it provided them the opportunity to feel the presence of their family members and feel as if they were getting together with their families. Baldassar [26] notes the following (page 161):

This might be defined as an ideal of shared co-presence, where the ideal of being there for each other is met with the actual practice of being in touch and the feeling of being in touch enough…Despite the potential pitfalls, the conditions of polymedia appear to provide the capacity to deliver a sense of adequate “distant co-presence” and adequate “distant care.”

These descriptions are consistent with previous studies on refugees’ use of ICTs [14,15,25,38]. Communication among separated family members also provides refugees with a coping mechanism for the adversities they face in their adopted countries [39]. Copresence, the idea of being emotionally present with family, through the use of ICTs, has recently received much attention from researchers [18,26,39,40]. Baldassar argues that communication among separated family members increases the urge to be physically present with one’s family members. Among immigrants who are able to travel freely back to their home countries, they are able to fulfill their desire to be physically present with family members and are thus able to both physically and emotionally decrease the distance between themselves and their families.

However, in the case of most refugees, the inability to return home, whether because of ongoing conflict in their home country or policy restrictions that impede travel from their resettlement country, may result in communication through ICTs causing emotional distress. Unlike other documented immigrants who can travel to their home countries by choice, refugees often cannot visit their parents or other family members. They have been forced out of their countries; thus, their urge to be physically present with their family members can increase stress and depression.

In addition, our findings highlight the constraints on refugee agency in the use of ICTs. Although some refugees managed to take refuge in safe countries, others remained in conflict areas. For some participants, communication through the use of ICTs was keeping the memories of the trauma they had been through alive. Their communication was mostly about war and the fear of losing a family member to that war. Refugees employed agency in their choice of ICT channels to communicate with their families and friends. For example, some of our participants narrated how they did not use the *video chat* option while talking to their families to hide their adverse conditions from the families, but they could not control the messages they received from their friends and families. The exchange of messages with their family members was a frequent reminder of the traumatic conditions their families continued to experience, and this kept them immersed, albeit at a distance, in the difficulties they had fled.

Participants also discussed the ways in which ICTs could not replace face-to-face or physical interaction with their families. For example, refugees described how they could not express their feelings of agony and helplessness to their families through ICTs. Baldassar [26] argues that ICTs leave little excuse for not being there emotionally for each other: “the form of co-presence that the constant and varied uses of ICTs deliver has become an expected feature of daily life” (page 156). However, the constant presence of ICTs can also create confusion among separated family members. For example, a person can be online on WhatsApp or Viber 24 hours a day, but he or she might not be able to attend a call or reply to a message because of different reasons, such as a difference in the time zones or obligations, for example, work or school. A participant noted that communicating through ICTs increased the emotional distance between her husband and his family members, as their families could not understand how limited her husband’s time was for communication and as they were not able to understand how busy her husband was in the way they would be if they were in the same geographic locale. The lessening of communication within the family, despite the convenience of ICTs, had an adverse impact on both parties involved in the act of communication.

We found that financial and logistical issues also limited refugees’ ability to communicate with their families through ICTs. Some previous studies have noted that not all refugees have equal access to digital technology [13,28,33]. We did not specifically ask participants about accessing ICTs, but in response to open-ended questions about communication with family and friends, 9 participants talked about difficulties accessing ICTs. A total of 3 participants contended that they did not have enough financial resources to buy mobile phone calling cards, 2 participants said that they were not technologically literate, and 4 participants noted poor ICT infrastructure in their home countries.

### Recommendations or Practical Implications

Our study makes significant contributions to the understanding of communication through ICTs for separated refugee families. Our findings suggest some ways that organizations serving refugees in host countries may be able to use ICTs to promote the mental well-being of refugees. First, host countries could arrange tailored ICT usage training for refugee families. These trainings could include teaching them the use of different ICTs. These types of training could help bridge the digital literacy divide among refugees. In addition, refugee service providers and refugees themselves could be made aware of both the potential benefits and challenges of frequent communication with family members through the use of ICTs.

Host organizations should also understand the importance of helping recently arrived refugees access the internet or mobile services, at least during the early phases of their resettlement. The provision of free internet or mobile phones would enable refugees to communicate with their families. The provision of ICTs could also help refugees better integrate into their host countries. As some previous studies have shown, refugees not only use ICTs to communicate with their families but also use these tools to access different social services and learn about the political and cultural environments of their host countries [25,30,31]. The development of a mobile app specifically for refugees might also be helpful. The mobile app could contain features such as social support, information about their new country, and how to get access to different social services. Mobile apps should be created in the languages of refugees. The apps should also have audio and video features. The audio and video features could help refugees whose literacy rates are low and who cannot read in their native languages.

### Limitations

Even though our study makes a critical contribution to the existing literature on refugee communication, it has a few limitations. We were able to explore how ICTs affected participants’ mental health. However, as we did not specifically and systematically ask refugees about their use of ICTs, we were not able to compare whether refugees with different background characteristics were more likely to have positive or negative experiences with the use of ICTs in terms of the impact on their mental health. Future researchers could use quantitative tools to investigate the relative impact of ICTs on people of different backgrounds and social locations. Another notable limitation is that our study design focused solely on the use of ICTs among refugees for communication with their separated families. Earlier research shows that refugees also use ICTs to get news about their host countries and their country of origin. Obtaining information through different news websites can also affect refugees’ mental health in several ways. Future research could test the impact of Web-based information available to refugees on their well-being. We also did not measure whether age, sex, or education level had any effect on the medium of choice of the participants—such as Facebook, Skype, WhatsApp, Viber, and Mobile phone. Future research could also test or explore whether the type of ICT platform explains any variance in refugees’ mental health.

### Conclusions

ICTs play a critical role in bridging the communication gap among separated refugee families. This study sought to explore how refugee adults from Afghanistan, Iraq, Syria, and the Great Lakes region of Africa (Burundi, Democratic Republic of Congo, and Rwanda), who were resettled in the United States for less than 3 years, perceived the effect of ICT communication with their separated families on their mental health and well-being. We found that the use of ICTs has both positive and negative impacts on the mental health of refugees and that refugees recognize this and thus make strategic decisions about how much, when, and which ICT to use to communicate with distant family members. Several refugees in our study also faced strategic and financial problems in communicating with their separated families through ICTs. These findings highlight the complexity of ICT use among refugees, both in terms of how it can ease or exacerbate the distress caused by family separation for people who are forcibly displaced and in terms of inequities in access to ICTs.

## Abbreviations

ICTs: information and communication technologies
NET: Narrative Exposure Therapy
PTSD: posttraumatic stress disorder
RWP: Refugee Well-being Project
UNHCR: United Nations High Commission for Refugees
